# The science of electrical stimulation therapy for fracture healing

**DOI:** 10.4103/0019-5413.50846

**Published:** 2009

**Authors:** Paul RT Kuzyk, Emil H Schemitsch

**Affiliations:** Department of Surgery, University of Toronto, Toronto, ON, Canada

**Keywords:** Electrical stimulation, fracture healing, direct electrical current, capacitive coupling, inductive coupling

## Abstract

This article is a brief review of the basic science research conducted in the field of electrical stimulation for fracture healing. Direct electrical current, capacitive coupling, and inductive coupling have been studied as potential techniques to enhance fracture healing through the proliferation and differentiation of osteogenic cells. These techniques are particularly appealing as they offer a potential minimally invasive solution to the difficult clinical problem of delayed fracture healing and nonunion. Basic science studies have shown conclusively that electrical stimulation techniques lead to bone cell proliferation and have attempted to elucidate the intracellular processes by which this bone cell proliferation occurs. Further basic science and clinical research is required to enhance the effectiveness of this therapy for the treatment of fracture nonunions.

## INTRODUCTION

The relationship between physical forces and bone biology has been recognized since the early 1800s.^1^,^2^ Mechanical forces (compression, distraction, and shear), electrical forces, magnetic forces, and ultrasonic waves have all been found to exert some level of effect on bone growth and healing.^3^–^6^ Electrical stimulation of bone has been touted as an effective and noninvasive method for enhancing bone healing, and treating fracture nonunion. Unfortunately, clinical evidence for the efficacy of electrical stimulation is limited. A recent meta-analysis by Mollon *et al.* could only identify four randomized controlled trials evaluating the clinical use of electrical stimulation to treat delayed union and nonunion of fractures.^7^ Despite the lack of clinical evidence, many *in vitro* and *in vivo* studies demonstrate the usefulness of electrical stimulation in bone healing at a cellular level. Furthermore, these studies provide us with an understanding of the underlying cellular mechanism by which electrical stimulation influences fracture healing.

## BASIC SCIENCE OF ELECTRICAL STIMULATION OF BONE

The mechanical stress applied on bone results in the generation of electrical potentials.^8^,^9^ Electronegative potentials are generated with compression and electropositive potentials are generated with tension. Piezoelectric properties of the collagen matrix and electrokinetic effects (or streaming potentials) cause these electric potentials in response to the mechanical environment.^10^ It has been shown that bone is formed under electronegative potentials and resorbed under electropositive potentials.^11^ It is thought that this electrical stimulation is the path through which bone forms in response to applied load.

The observation regarding the electrical nature of bone osteogenesis has spurred the development and investigation of techniques for applying electrical fields to fracture sites in an effort to promote healing. Three techniques for the application of electrical stimulation in fracture healing have been described, which include direct electrical current, capacitive coupling, and inductive coupling [Table 1].

**Table 1:** A summary of the techniques of electrical stimulation of bone

Technique of electrical stimulation	Method of application	Advantages	Disadvantages
Direct electrical stimulation	One or multiple surgically implanted cathodes with one cutaneous electrode	Case series (Level IV) suggests clinical efficacy^13^ May enhance growth factor production	Invasive (requires surgical implantation of cathodes)
Capacitative coupling	Two cutaneous electrodes	Noninvasive Basic science studies show enhanced bone cell proliferation May enhance growth factor production	
Inductive coupling	Cutaneous electromagnetic coil	Noninvasive Basic science studies show enhanced bone cell proliferation May enhance growth factor production	Recent meta-analysis (Level I) failed to show clinical efficacy^7^

Electrical stimulation techniques have been applied to acute fractures, delayed unions, nonunions, and joint arthrodesis. Contraindications to electrical stimulation include segmental bone loss at the fracture site, synovial pseudoarthrosis, congential pseudoarthrosis, infected nonunions, and poor mechanical stability of the fracture site. In these clinical scenarios, surgical management to bone graft defects, eradicate infection, or stabilize the fracture with internal fixation is required before electrical stimulation can be considered. Electrical stimulation should be thought of as an adjunct to, not a replacement for, standard fracture care. We will examine each of the three methods of electrical stimulation in detail.

## DIRECT ELECTRICAL CURRENT

**Figure 1:**
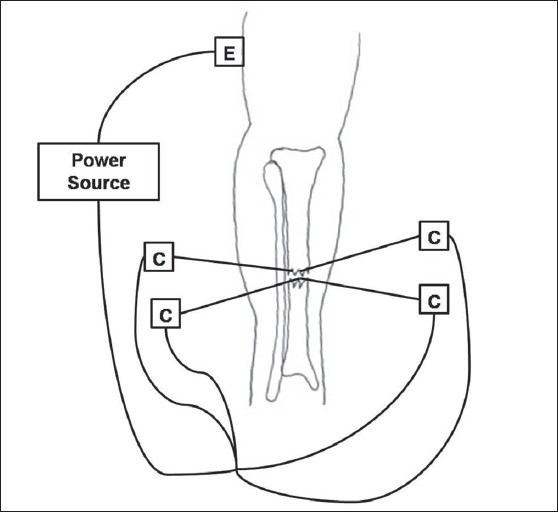
The technique of direct electrical stimulation of bone is illustrated for a tibia fracture. Four cathodes are implanted at the fracture site and a cutaneous electrode is placed at a distant site. An external power source is used to generate current. E = Electrode, C = Cathode

Direct electrical current techniques are invasive and involve the implantation of one or multiple cathodes into the bone [Figure 1]. An anode is typically placed on the skin over the fracture site and a 5 to 100μA current is delivered.^12^ In 1981, Brighton *et al.* published a case series using direct electrical stimulation via four cathodes surgically implanted into a fracture nonunion site for 12 weeks (Level IV Evidence).^13^ They found that four 20-μA cathodes applied for 12 weeks produced solid bony union in 129 of 168 fracture nonunions (i.e., 76.8% union). The authors suggested that the presence of a synovial pseudoarthrosis, a large bone gap at the fracture site, or an osteomyelitis were contraindications to electrical stimulation therapy and therefore removed these patients from their clinical series. Direct electrical current has also been used to promote healing of spinal fusion, ankle fusions and charcot foot reconstructions.^14^–^16^

An electrochemical reaction occurring at the cathode is thought to, in part, result in the osteogenic effects of direct electrical stimulation. A faradic reaction at the cathode has been shown to lower oxygen concentration, increase pH, and produce hydrogen peroxide.^17^ Such a decrease in oxygen concentration has been found to enhance osteoblastic activity, whereas basic environments have been shown to both increase osteoblastic activity and decrease osteoclastic activity.^18^ The direct electrical current also results in increased proteoglycan and collagen synthesis. In addition, hydrogen peroxide may stimulate macrophages to release vascular endothelial growth factor (VEGF), an angiogenic factor that is critical for osteogenesis.^19^,^20^

## CAPACITIVE COUPLING

**Figure 2:**
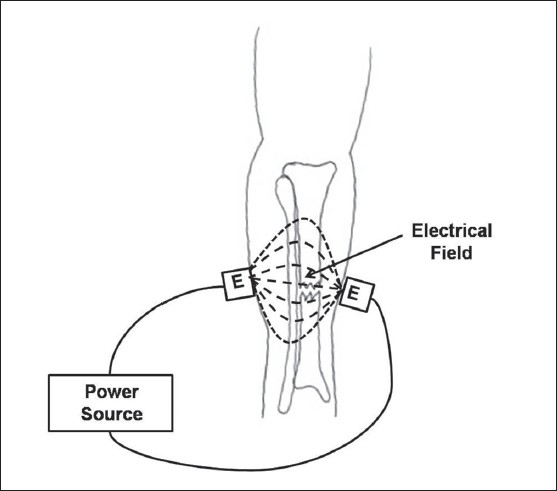
The technique of capacitative coupling is illustrated for a tibia fracture. Two coupled electrodes are placed on the skin overlying the fracture site and an external power source is used to generate current. An electrical field is produced between the electrodes and through the fracture site. E = Electrode

Capacitive coupling is a noninvasive technique that involves placing two electrodes on the skin overlying the fracture such that the fracture site lies between the electrodes [Figure 2]. An alternating current is then used to create an electrical field within the fracture site. Potentials of 1–10 V at frequencies of 20–200 kHz are applied to the electrodes, which result in the development of electric fields of 1–100 mV/cm at the fracture site.^21^

Brighton *et al.* found that the electrical field strength played a major role in determining the proliferation of bone cells when exposed to a capacitive coupling electric field.^22^ Stimulation of proliferation of rat calvarial bone cells was measured by 3H thymidine incorporation into DNA and alkaline phosphatase production. They found that an electrical field strength of 0.1–10 mV/cm induced proliferation of rat calvarial bone cells, and electrical field strengths less than 0.1 mV/cm did not induce proliferation.

Korenstein *et al.* found that there was a dose-dependent response to capacitive coupled fields whereby greater electrical field strength leads to greater proliferative response in osteoblast cells.^23^ An increase in the time the bone cells are exposed to the electrical field (or “duty cycle” of the capacitive coupling) has also been shown to increase bone cell proliferation.^24^

**Figure 3:**
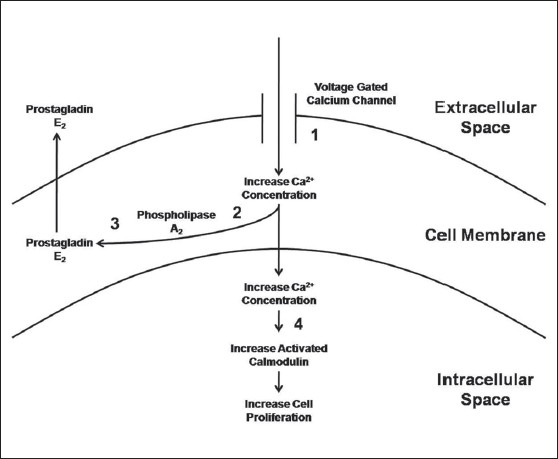
Schematic drawing depicting the signal transduction pathway followed by capacitive coupling electrical stimulation. Numbers represent the inhibitor that blocks the associated pathway: 1 = verapamil, 2 = bromophenacyl bromide, 3 = indomethacin, and 4 = W-7. (Adapted from Brighton C, Wang W, Seldes R, Zhang G, Pollack S: Signal transduction in electrically stimulated bone cells. J Bone Joint Surg. 2001; 83A:1514-1523.)

The chemical pathway by which capacitive coupling acts on the bone cell to cause proliferation and osteogenesis is a matter of current study.^25^ Bone cell proliferation resulting from capacitive coupling is accompanied by an increase in intracellular calcium concentration. It has been shown that the proliferative response of bone cells to a capacitive coupling is mediated by calcium translocation via voltage-gated calcium channels. Lorich *et al.* found that verapamil (a voltage-gated calcium channel blocker) halted the bone cell proliferation seen with capacitive coupling, whereas neomycin (a blocker of the inositol phosphate pathway) had no effect on this proliferation.^26^ A further study revealed that bromophenacyl bromide (an inhibiter of phospholipase A), indomethacin (an inhibiter of prostaglandin synthesis), and W-7 (a calmodulin antagonist) has a similar effect on bone cell proliferation.^27^ These studies suggest that, for capacitive coupling, signal transduction results from calcium ion translocation through voltage-gated calcium channels that leads to increases in prostaglandin, cytosolic calcium, and activated calmodulin [Figure 3].

Wang *et al.* found that capacitive coupling up-regulates the mRNA expression for bone morphogenic proteins (BMPs)-2, -3, -4, -5, -6, -7, and -8, as well as gremlin and noggin.^24^ This increase in the production of growth factors that are important for the proliferation and differentiation of osteoblastic cells may represent an alternative mechanism by which capacitive coupling influences osteogenesis.

## INDUCTIVE COUPLING

**Figure 4:**
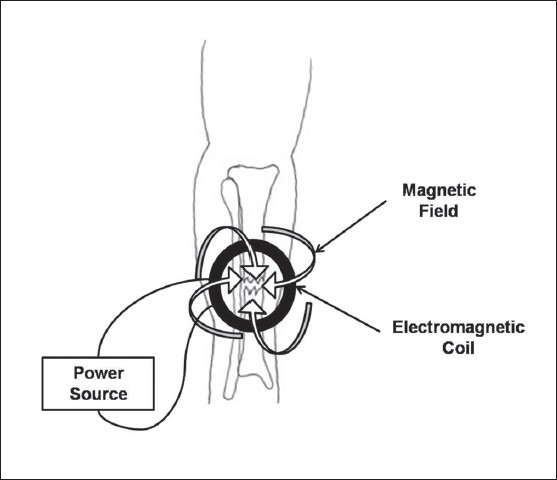
The technique of inductive coupling is illustrated for a tibia fracture. An inductively coupled electromagnetic coil is placed on the skin overlying the fracture site. An external power source produces a circular current within the coil which produces a magnetic field perpendicular to the direction of the current. This magnetic field induces an electrical field within the fracture site

The third type of electrical stimulation used to enhance fracture healing is inductive coupling. Inductive coupling relies on the use of a pulsed electromagnetic field (PEMF) device that is placed on the skin over the fracture site [Figure 4]. The PEMF consists of a wire coil through which a current is passed and a magnetic field is generated. The magnetic field, in turn, induces an electrical field within the fracture site. The size of the electrical field that is induced within the fracture site is dependent on the magnitude of the magnetic field and the physical characteristics of the tissues surrounding and within the fracture site. The variability of the current flowing through the PEMF results in an induced magnetic field that is time variable, and thus the magnitude of an electrical field within the bone varies with time. Induced magnetic fields varying from 0.1 to 20G have been used to produce electrical fields varying from 1 to 100 mV/cm within bone.^28^ This time-varying electrical field is thought to simulate the normal response of bone cells physiologically to applied mechanical stress.^29^

**Figure 5:**
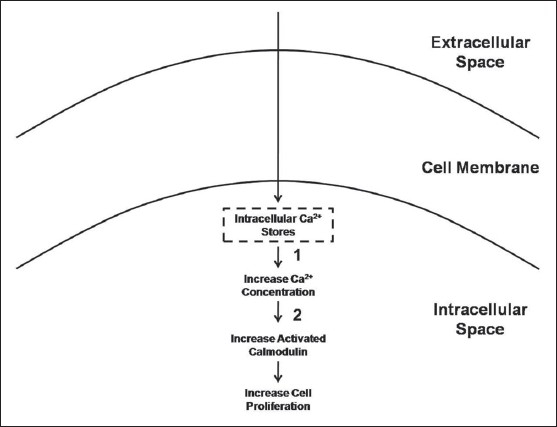
Schematic drawing depicting the signal transduction pathway followed by inductive coupling electrical stimulation. Numbers represent the inhibitor that blocks the associated pathway: 1 = TMB-8 and 2 = W-7. (Adapted from Brighton C, Wang W, Seldes R, Zhang G, Pollack S: Signal transduction in electrically stimulated bone cells. J Bone Joint Surg 2001;83A:1514-1523)

Interestingly, inductive coupling results in an increased bone cell proliferation with increased cytosolic calcium concentration similar to that seen with capacitive coupling. However, the chemical pathway is different. The increase in bone cell proliferation with inductive coupling was blocked by TMB-8 (blocks release of intracellular calcium) and W-7 (blocks activation of calmodulin), but not with the inhibitors found to block bone cell proliferation encountered with capacitive coupling (verapamil, bromophenacyl bromide, or indomethacin).^24^ For bone cell proliferation due to inductive coupling, signal transduction must be mediated by release of intracellular calcium leading to increases in cytosolic calcium and activated calmodulin [Figure 5].

Similar to capacitive coupling, studies have shown increases in growth factors with the use of inductive coupling. BMP-2 and BMP-4 mRNA expression were found to be significantly increased in chick osteoblasts after undergoing inductive coupling.^30^ Another study cultured nonunion cells from fracture nonunion patients and found a significant increase in TGF-β (Transforming Growth Factor Beta) production in cells stimulated with inductive coupling versus control cells.^31^ This may represent an alternative method whereby inductive coupling may influence proliferation and differentiation of osteoblastic cells.

A meta-analysis by Mollon *et al.* examined four randomized controlled trials evaluating the clinical use of inductive coupling electrical stimulation to treat delayed union and nonunion of fractures.^7^ Their meta-analysis revealed high heterogeneity (I^2^ = 60.4%) that could not be explained by the bone investigated (tibia versus other bone) or the lesion treated (delayed union versus nonunion). They suggested that this heterogeneity may be due to the varied treatment devices and treatment times used in the different studies. The authors concluded that current evidence from randomized clinical trials is insufficient to suggest a clinical benefit for the use of this therapy for fresh fractures, osteotomies, delayed unions, and nonunions (level I evidence).

## SUMMARY

There is extensive basic science research published on the effects of electrical stimulation for fracture healing. These studies have examined the effects of direct electrical stimulation, capacitive coupling and inductive coupling on bone cells *in vitro*. Through this research, we are now beginning to understand the mechanism of action of these modalities at the cellular level. Further research is required to better elucidate the chemical pathways within the bone cell that respond to electrical stimulation and result in proliferation and differentiation into osteoblastic cells. This may allow us to improve the effectiveness of electrical stimulation for enhancement of fracture healing and treatment of fracture nonunion in humans.
